# Die „gesunde Kommune“ im Lichte „großer Wenden“ – ein sozialökologisch fundiertes Ziel kommunaler Gesundheitsförderung (KoGeFö)

**DOI:** 10.1007/s11553-021-00889-y

**Published:** 2021-08-10

**Authors:** Wolfgang Schlicht, Jens Bucksch, Carl-Walter Kohlmann, Britta Renner, Jürgen Steinacker, Fabian Walling

**Affiliations:** 1 Fakultät für Wirtschafts- und Sozialwissenschaften, Universität Stuttgart, Taubenstraße 46, 72108 Rottenburg, Deutschland; 2 Pädagogische Hochschule Heidelberg, Heidelberg, Deutschland; 3 Pädagogische Hochschule Schwäbisch Gmünd, Schwäbisch Gmünd, Deutschland; 4 Universität Konstanz, Konstanz, Deutschland; 5 Universitätsklinikum Ulm, Ulm, Deutschland; 6 Hochschule für Verwaltung und Finanzen Ludwigsburg, Ludwigsburg, Deutschland

**Keywords:** Intersektorale Gesundheitsförderung, Resilienz, Fairness, Möglichkeitsräume, Verwirklichungschancen, Intersectoral health promotion, Fairness, Opportunities, Capabilities, Resilience

## Abstract

**Hintergrund:**

In Kommunen wird die Gesundheit der Bewohner*innen durch Lebensumstände geschützt, gefördert oder gefährdet. Kommunale Gesundheitsförderung (KoGeFö) findet in und mit der Kommune statt. In der Kommune konzentrieren sich Programme und Maßnahmen auf Endpunkte der Morbidität und Mortalität. Die Krankheitslast soll reduziert, sowie die individuelle Lebensqualität gestärkt werden. Mit der Kommune will Gesundheitsförderung die „gesunde Kommune“ entwickeln.

**Fragestellung:**

Wann ist eine Kommune „gesund“? Welche Absichten werden in der Gesundheitsförderung mit der Kommune jenseits von Programmen verfolgt, die auf die Reduktion der Inzidenz und Prävalenz nicht-ansteckender Erkrankungen zielen, indem sie die Bewohner*innen motivieren und unterstützen, sich gesundheitsfördernd zu verhalten?

**Material und Methoden:**

Vor dem Hintergrund „großer gesellschaftlicher Herausforderungen“ und mit Rückgriff auf sozialökologische Ansätze wird erörtert, was eine „gesunde Kommune“ ausmacht, worauf die Gesundheitsförderung mit der Kommune zielt.

**Ergebnisse:**

Die „gesunde Kommune“ entwickelt sich in der intersektoralen Zusammenarbeit von Akteur*innen der Politik, von Verwaltungseinheiten, der Zivilgesellschaft und der Bewohner*innen. Die „gesunde Kommune“ ist als faire Umgebung gestaltet. Sie öffnet den Einzelnen Möglichkeitsräume für dessen Handeln und gewährt Verwirklichungschancen für persönlich wichtige Ziele.

**Schlussfolgerung:**

Die bevorzugte sozialökologische Perspektive schärft den Blick für die dynamische Interaktion von Umwelt- und Personenfaktoren. Mit Fairness, Möglichkeitsräumen und Verwirklichungschancen sind drei Kriterien benannt, die sich als Gradmesser für den Endpunkt „gesunde Kommune“ einer Gesundheitsförderung mit der Kommune eignen.

## Abstract (en)

**Background:**

Communities are environments which can protect and promote or, conversely, can endanger the health of their members. Community health promotion takes place *in* and *with* a community. Implementing programs and measures *in* a community mainly focus on individuals and reducing endpoints such as morbidity and mortality, as well as increasing the quality of life. Extending this individual-focused approach, health promotion *with* a community aims at developing a “healthy community”.

**Objectives:**

What does health promotion with the community aim at? When is a community “healthy”? Beyond a reduction of endpoints such as the incidence and prevalence of nonommunicable diseases, what is the goal of programs, measures, and activities to motivate and support residents to adopt health-promoting behaviors?

**Materials and methods:**

Against the backdrop of “grand societal challenges” and drawing on socioecological approaches, the paper discusses what constitutes a “healthy community” and what health promotion *with* the community aims to achieve.

**Results:**

A “healthy community” develops through cooperation of multiple stakeholders from different sectors such as politics, administration, civil society, and residents. The “healthy community” is designed as a fair environment. It opens up possibilities for the individual to act and provides opportunities for them to pursue personally important goals.

**Conclusions:**

The socioecological perspective sharpens the view for the dynamic interaction of environmental and individual factors. Fairness, spaces of opportunity, and realization chances are three criteria that are suitable as indicators for a “healthy community”.

Kommunale Gesundheitsförderung gilt als wirksame Public-Health-Strategie. Sie adressiert die Bewohner*innen, Settings in der Kommune (z. B. Schulen) oder die Kommune als Lebenswelt. Sie will die „individuelle Resilienz“ stärken, indem sie Risikofaktoren (z. B. Übergewicht, chronischen Stress) der somatischen (z. B. Diabetes) und psychischen Gesundheit (z. B. Depressivität) reduziert. Sie strebt nach „gemeindlicher Resilienz“, indem sie die „gesunde Kommune“ entwickeln will. Die „gesunde Kommune“, wird angesichts von Klima-, demografischem Wandel und anderen „Großen Herausforderungen“ wichtiger.

## Kommunen als gesunde Lebenswelten

In Dörfern und Städten wachsen Menschen auf. Dort lernen, arbeiten, lieben, streiten, altern und sterben sie, nehmen am sozialen Leben teil, streben danach, ihre Bedürfnisse nach sozialem Anschluss, persönlichem Wachstum und Autonomie zu befriedigen. In Kommunen werden Gesundheit und Wohlbefinden gefährdet, gesichert und gefördert [4, 14]. Kommunalpolitische Entscheidungen und kommunales Verwaltungshandeln betreffen das Leben der Bewohner*innen unmittelbar.

Kommunen sind von Prozessen herausgefordert, unter denen Schneidewind [31] „sieben Wenden“ zur „gesellschaftlichen Transformation zur Nachhaltigkeit“ als dringlich hervorhebt. Darunter sind nicht zuletzt die Konsum-, die Mobilitäts-, die Ernährungs- und die urbane Wende für die kommunale Gesundheitsförderung (KoGeFö) relevant. Der Wissenschaftsrat [42] hält nachhaltige Transformationen in Folge der „großen gesellschaftlichen Herausforderungen“ für geboten und der Wissenschaftliche Beirat der Bundesregierung Globale Umweltveränderungen (WBGU) stellt in seinen Gutachten auf die gesundheitlichen Folgen von Transformations- und Urbanisierungsprozessen ab [36, 37]. Neben ökologischen sind zudem sozialbedingte Herausforderungen mit der Bevölkerungsgesundheit assoziiert [29]. Auch im Aktionsprogramm „Agenda 2030“ der UN, das 17 Nachhaltigkeitsziele benennt, wird gefordert, dass Kommunen an nachhaltigen Transformationen mitwirken sollen, um die Bevölkerungsgesundheit zu sichern [33].

Kommunen können nicht zuletzt mitwirken, weil sie mit ihren prinzipiell flexiblen Verwaltungsstrukturen direkt auf die Konsequenzen der gesellschaftlichen Herausforderungen antworten können. In Vereinen, Selbsthilfegruppen und anderen Gemeinschaften – wie auch bei kommerziellen Dienstleistern – organisieren sich zudem bereits erprobte zivilgesellschaftliche Aktivitäten. Anders als die Bundesländer oder der Bund können Kommunen ihre Bewohner*innen dauerhaft direkt und nicht nur wiederkehrend und repräsentativ an Entscheidungen beteiligen. Mit Gesundheits- und Sozialämtern verfügen sie über Verwaltungseinheiten, die sich mit gesundheitlichen Belangen befassen und (mancherorts) mit „kommunalen Gesundheitskonferenzen“ auch über partizipative Formate. Das alles lässt sich nutzen, um die Gesundheit der Bewohner*innen vor dem Hintergrund der Transformationserfordernisse bedarfsorientiert und zielgerichtet zu sichern und zu fördern. KoGeFö gilt gerade wegen der Bürger*innennähe als potenziell wirksame Public-Health-Strategie [3, 14]. Gleichwohl fehlt es neben der argumentativen Plausibilität des Potenzials der KoGeFö noch an empirischer Evidenz zur Wirksamkeit. Der fehlende Nachweis der Wirksamkeit ist nicht zuletzt der schwierigen Bestimmung von Adressat*innen und der Indikatoren für „outcomes“ und „impacts“ bei komplexen populationsbasierten Interventionen mit niedriger Effektstärke geschuldet.

Die KoGeFö adressiert mit Programmen, Maßnahmen und Aktivitäten drei Zielobjekte („targets“): (1) die Kommune als Lebenswelt (z. B. die aktivitätsfördernde oder generationengerechte Quartiersentwicklung), (2) Settings in der Kommune (z. B. Betriebe, Kindergärten, Schulen) und (3) das Verhalten der Bewohner*innen (z. B. die körperliche Aktivität). Sie zielt zum einen auf die „individuelle“ und zum anderen auf die „gemeindliche Resilienz“. Im Beitrag konzentrieren wir uns auf die Kommune als Lebenswelt, damit auf die „gemeindliche Resilienz“ und nennen diese Variante der KoGeFö – angelehnt an Boutillier, Cleverly und Labonte [6] – Gesundheitsförderung *mit* der Kommune. Die beiden anderen Varianten betreffen die KoGeFö *in* der Kommune.

## Gemeindliche Resilienz: eine Annäherung

Mit dem Ziel der „Resilienz“ hat die WHO [39] ihre in der Ottawa-Charta und in nachfolgenden Deklarationen formulierten Strategien des gesundheitspolitischen Handelns bestärkt: Die Bevölkerung soll vor gesundheitlichen Risiken geschützt, Gesundheit und Wohlbefinden sollen gefördert und das Gemeinwesen soll gegenüber zukünftigen Bedrohungen robust gemacht und befähigt werden, selbstbestimmt zu handeln.

„Resilienz“ wird je nach wissenschaftlicher Disziplin unterschiedlich definiert und gebraucht. „Individuelle Resilienz“ wird noch am ehesten einheitlich, als das Vermögen einer Person verstanden, sich nach kritischen Lebensereignissen (z. B. Misshandlungen in der Kindheit) zu erholen und die Fähigkeit aufrecht zu erhalten, trotz erfahrener widriger Bedingungen, zu gesunden. „Individuelle Resilienz“ gleicht den „generalisierten Widerstandsressourcen“ im „Salutogenesemodell“ von Antonovski [1]. Hobfoll [17] – von diesem Modell inspiriert – betont neben persönlichen (Eigenschaften und Fähigkeiten) auch Objekt- (z. B. ein Haus zu besitzen), Bedingungs- (z. B. ein herausgehobener Berufsstatus) sowie Energieressourcen (z. B. Verfügbarkeit über Zeit, Wissen, finanzielle Mittel), die für die Fähigkeit maßgeblich seien, Herausforderungen und Bedrohungen zu bewältigen.

Die „gemeindliche Resilienz“ ist unscharf definiert. Debatten zum Konstrukt speisen sich häufig aus der Perspektive der „Katastrophenforschung“ [21, 27]. Gemeinden (Dörfer und Städte) sind komplexe Sozialsysteme. Das Verständnis „gemeindlicher Resilienz“, das sich in das sozialökologische Paradigma fügt – das im Folgenden die Argumente fundiert – rekurriert auf „ecological resilience“, die grundlegend mit den Arbeiten von Holling [18] verknüpft ist. „Gemeindliche Resilienz“ umfasst drei Eigenschaften: (a) das Maß der Veränderungen, das eine Gemeinde tolerieren kann, ohne ihre Strukturen und Steuerungsfunktionen einzubüßen (z. B. durch soziale Verwerfungen angesichts einer Pandemie), (b) den Grad der Selbstorganisation kommunaler Gremien und der kommunalen Gemeinschaft, um den Status quo (ante) aufrecht zu erhalten respektive wiederherzustellen und (c) die Fähigkeit, Herausforderungen mit Wandel zu begegnen, um Identität, Funktionen, Strukturen und soziale Rückkopplungsprozesse zu bewahren (adaptieren und lernen). Das Bewahren und das Wiederherstellen also, aber auch die Wandlungsfähigkeit sind inhärente Eigenschaften „gemeindlicher Resilienz“ [11].

Aus sozialökologischer Perspektive ist zu bedenken, dass Kommunen mit unter- und übergeordneten Einheiten verknüpft sind, in denen eigene „adaptive Zyklen“ ablaufen, die zur Resilienz führen. Nachbarschaften, Quartiere, Dorf, Gesamtstadt und Landkreis sind „panarchisch“ verbunden. Mit einem unterschiedlichen Grad an Beeinflussbarkeit ihrer natürlichen, sozioökologischen, ökonomischen und kulturellen Eigenschaften und je nach Verfügbarkeit an materiellen und sozialen Ressourcen sind die unter- und übergeordneten Einheiten an der Transformation des Gesamtsystems beteiligt. KoGeFö *mit* der Kommune kann in der Nachbarschaft ansetzen. Sie kann einzelne Quartiere in den Blick nehmen und muss nicht notwendiger Weise die Stadt oder das Dorf als Ganze(s) adressieren. Was sich in einer Untereinheit (z. B. Nachbarschaft) wandelt, das berührt die übergeordnete Einheit (z. B. Quartier) und was sich übergeordnet tut, wirkt auch untergeordnet.

## Kommunale Daseinsvorsorge

Kommunen obliegt die „Daseinsvorsorge“ ihrer Bewohner*innen. Dazu bilden das „Sozialstaatsprinzip“ (Art. 20 I GG) sowie die „Garantie der kommunalen Selbstverwaltung“ (Art. 28 II GG) die verfassungsrechtlichen Grundlagen. Die Leistungspflichten der Kommunen – mit Bezug zur individuellen Gesundheit – speisen sich aus weiteren Prinzipien, wie der „Menschenwürde“ (Art. 1, I GG) und dem „Recht auf Leben und körperliche Unversehrtheit“ (Art. 2, II 1 GG). Kommunales Handeln erbringt mindestens Leistungen, „derer der Bürger zur Sicherung einer menschenwürdigen Existenz unumgänglich bedarf“ (BVerfG, Urteil vom 20. März 1984, Az. 1 BvL 28/82, Leitsatz = BVerfGE 66, 248, 258).

Was bedeutet „Daseinsvorsorge“ in der Praxis? Kommunales Handeln adaptiert an potenzielle Umweltbedrohungen (Adaptation), gestaltet die natürliche, soziale, gebaute, ökonomische und technische Umwelt vorsorgend gegen mögliche Bedrohungen (Mitigation). Wegen der Unbestimmtheit des Rechtsbegriffs „Daseinsvorsorge“ legt jede Kommune – im Rahmen der geltenden Gesetze – fest, was sie an adaptiven und/oder gestalterischen Aufgaben lösen will. Allgemein gehören verschiedene Aufgabenfelder dazu: Energie- und Wasserversorgung, Abwasser- und Abfallentsorgung, Polizei und Feuerwehr, Altenpflege, Friedhöfe, sozialer Wohnungsbau, gesundheitliche Versorgung, öffentlicher Personennahverkehr, Wirtschaftsförderung sowie Einrichtungen, die sich für kulturelle (Bildungseinrichtungen), sportliche und soziale Aktivitäten nutzen lassen.

Was eine Kommune an Aufgaben bearbeitet, ist – neben den gesetzlichen Vorgaben – vom politischen Wollen der kommunalen Entscheider*innen (Ortsvorsteher*in, Bürgermeister*in, Landrät*in) und Gremien (Bezirksversammlung, Gemeinderat, Kreistag) abhängig. Gesundheitsförderung steht nicht zwingend auf der Agenda. In der Pflicht zur kommunalen „Daseinsvorsorge“ wird sie auch nicht explizit als Aufgabe benannt. Eher wird die Sicherung der Gesundheitsversorgung als kommunale Aufgabe begriffen [35], obgleich sie nur mittelbar in die Entscheidungshoheit von Kommunen fällt (z. B. die Sicherung der ambulanten Versorgung in einem Dorf oder die stationäre Versorgung im Landkreis).

## Gesellschaftliche Herausforderungen und sozialökologische Transformation

Misslingen angesichts der „großen gesellschaftlichen Herausforderungen“ Adaptation und Mitigation, dann werden das soziale, das wirtschaftliche und das kulturelle Leben substanziell bedroht. Das System wird vulnerabel, instabil in seinen Strukturen und büßt sein Selbstorganisationsvermögen ein. Klima- und demografischer Wandel, Urbanisierung, Digitalisierung, automatisches Entscheiden, künstliche Intelligenz und weitere Herausforderungen drängen auf die resiliente Gestaltung der Kommune.

Die Vereinten Nationen haben angesichts der „großen gesellschaftlichen Herausforderungen“, die sich in der globalen Welt in unterschiedlichem Maße und mit unterschiedlicher Dringlichkeit manifestieren, 5 Dimensionen und 17 Ziele zur Nachhaltigkeit benannt, um die ökonomische, ökologische und soziale Entwicklung zukunftsfähig und generationsübergreifend zu sichern [33]. KoGeFö korrespondiert unmittelbar mit den Nachhaltigkeitszielen (Nr. 3) „ein gesundes Leben für alle gewährleisten und fördern“ und kann zu weiteren Zielen entscheidend beitragen: (Nr. 10) „Abbau von Ungleichheiten“, (Nr. 11) „Städte und Gemeinden robust und nachhaltig entwickeln“ und (Nr. 13) „Handeln für den Klimaschutz“.

Die „Herausforderungen“ für die Kommunen koinzidieren in der nahen Zukunft, beschleunigen und verstärken sich in ihren Folgen gegenseitig und bedingen den Verlust an natürlichen Ressourcen (z. B. Habitatzerstörung, Wasser- und Luftverschmutzung). Sie gefährden das Sozialkapital [10, 28], indem sie soziale Disparitäten verstärken (z. B. Zugänglichkeit zu „sauberen“ Umwelten).

Bedrohungen für das soziale und wirtschaftliche Leben resultieren zusätzlich aus der erhöhten Wahrscheinlichkeit pandemischer Ereignisse (z. B. SARS-CoV‑2 [„severe acute respiratory syndrome coronavirus 2“]). Die beeinträchtigen die Bevölkerungsgesundheit massiv (z. B. Long-COVID [„coronavirus disease“], Übersterblichkeit), offenbaren und verstärken soziale Ungleichheiten. Pandemische Ereignisse sind keine reinen, über die Gesellschaft schicksalhaft hereinbrechende, Naturgewalten. Pandemien resultieren u. a. aus Zoonosen, die durch naturzerstörende Lebensweisen (mit)bedingt sind und sich – gibt es keinen Wandel der Lebensweisen – zukünftig wahrscheinlicher wiederholen werden [20]. Brand und Wissen fordern eine radikale Umkehr von der „imperialen Lebensweise“ [7].

Sich vorsorgend auf Katastrophen vorzubereiten (z. B. durch Vorratshaltung medizinischer Güter), sich mit Maßnahmen des Infektionsschutzes (z. B. Surveillance) gegen gesundheitliche Bedrohungen zu wappnen, die aus den „Herausforderungen“ resultieren und daraus für zukünftige Herausforderungen zu lernen, ist „kommunale Daseinsvorsorge“. Kommunen sind einerseits „Verursacher“ und andererseits „Betroffene“ der „Herausforderungen“. Sie sind gefordert, die Lebensgrundlagen der Bewohner*innen und die Kommune als funktionierende Gemeinschaft zu sichern und sie im Lichte der Herausforderungen lernend weiter zu entwickeln.

Mehrere Kommunen sind in der „Agenda 21“ bereits dem Motto „Global denken – lokal handeln“ gefolgt, indem sie vier Dimensionen des nachhaltigen Wandels adressiert haben: Umwelt, Soziales, Kultur und Wirtschaft. Lokal haben sie Verantwortung für globale Prozesse übernommen, ihre Bewohner*innen beteiligt, Zielkonflikte ausgetragen (z. B. zwischen Umweltschutz und Wirtschaftswachstum) und politisches und Verwaltungshandeln mit Blick auf die „großen gesellschaftlichen Herausforderungen“ integriert. Auch auf den Politikfeldern Bildung, Wohnen, Klima und Energie sind Kommunen aktiv. Gesundheitsförderung wird dagegen noch selten als kommunales Politikfeld bearbeitet [https://www.bertelsmann-stiftung.de/de/unsere-projekte/agenda-2030-nachhaltige-entwicklung-vor-ort].

Die „Arbeitsgruppe Gesundheitsfördernde Gemeinde- und Stadtentwicklung“ beim „Deutschen Institut für Urbanistik“ hat 2020 fünf Thesen veröffentlicht, um Kommunen zur nachhaltigen KoGeFö anzuregen: (a) zunächst müssten sie wahrnehmen, dass sie von den „großen gesellschaftlichen Herausforderungen“ betroffen sein werden, dann sollten sie (b) Gerechtigkeit, Partizipation und Umweltschutz in den Blick nehmen und (c) integrierte Leitbilder, Handlungsansätze und Strategien entwickeln, um (d) in ihrer Infrastruktur wohnortnahe Dienste für Gesundheit, Pflege, Rekreation und nachhaltige Mobilität zu gestalten und schließlich (e) global denken [2]. Die Thesen sind plausibel, aber ohne eindeutigen Zielbezug: Welches Ziel (Outcome) verfolgt KoGeFö *mit* der Kommune?

## Gesundheitsförderung als kommunale Querschnittsaufgabe

Entscheidend für die weitere Debatte ist die eingangs getroffene Unterscheidung des Interventionsobjekts: KoGeFö *in* (Bewohner*innen; Settings) und KoGeFö *mit* der Kommune (Nachbarschaft, Quartier, Dorf oder Stadt, Landkreis).

Derzeit dominiert die KoGeFö *in* der Kommune. Sie adressiert das individuelle Verhalten und schafft in kommunalen Settings Bedingungen, die gesundheitsstärkendes und -förderndes Verhalten erleichtern (z. B. ein ausreichend hohes Volumen an körperlicher Aktivität). Das kann die Bevölkerung bereits zu einem nachhaltigeren Umgang mit natürlichen Ressourcen motivieren (z. B. reduzierter Fleischkonsum; verstärkte Nutzung des Fahrrads). Direktes Ziel ist es, die Inzidenz kardiometabolischer Erkrankungen und psychischer Störungen über Verhaltensänderungen (z. B. mehr körperliche Aktivität, weniger Fleisch- und mehr Gemüsekonsum) zu reduzieren. Richtet sich KoGeFö an Settings, um Gesundheitsverhalten zu erleichtern, nimmt sie räumlich verortete, kulturell geformte und zeitlich begrenzte Einheiten in den Blick, die mit ihren sozialen Interaktionen, dominierenden Werten und Einstellungen das Verhalten beeinflussen. Settings umfassen sowohl Organisationen (z. B. Betriebe, Kindergärten, Schulen) als auch Personen, die ein gleiches Merkmal tragen, ohne dass sie sich formal organisiert haben (z. B. alte alleinlebende Menschen; [8, 38]). Programme und Maßnahmen (u. a. von Krankenkassen auf der Grundlage des § 20 SGB V umfänglich angeboten), die auf die individuelle Resilienz zielen, sind gut begründet, adressieren aber nur zwei Adressaten der KoGeFö: die Bewohner*innen und die Settings *in* der Kommune (auch solche, die nicht im Entscheidungszugriff der Kommune stehen, wie etwa Betriebe oder kirchliche Einrichtungen).

Die KoGeFÖ *mit* der Kommune, die auf die „gemeindliche Resilienz“ zielt und die Kommune oder kommunale Untereinheiten (z. B. Nachbarschaften, Quartiere) adressiert, will mehr, als die Bewohner*innen schützen und ihr individuelles gesundheitliches Risiko mindern. Sie will die „gesunde Kommune“ entwickeln. Sie will in einem integrierten, partizipativen Ansatz das Sozialkapital der kommunalen Gemeinschaft stärken und die Gemeinde befähigen, Bedingungen zu schaffen, die das individuelle Wachstum der Bewohner*innen fördern, um ihnen ein „gutes Leben“ zu ermöglichen. Änderungen zielen systemisch auf die Identität, auf Strukturen und Funktionen der Kommune als komplexe Lebenswelt, in der gewohnt, gearbeitet und Freizeit verbracht wird (z. B. Transformation zur generationengerechten Kommune, Ermöglichung der gesundheitlichen Chancengleichheit etc.; [25]). Ansätze der KoGeFö *mit* der Kommune sind vor dem Hintergrund der „großen gesellschaftlichen Herausforderungen“ geboten, aber seltener erkennbar als Ansätze der KoGeFö *in* der Kommune. In der KoGeFö *mit* der Kommune stehen u. a. gesundheitliche Chancengleichheit, Partizipation, Umweltqualität, Klimaschutz, bewegungsförderliche Infrastruktur, Zugänglichkeit zu wohnortnahen Gesundheitsdiensten, Verfügbarkeit gesunder Nahrungsmittel oder Möglichkeiten zur Rekreation und Regeneration auf der politischen Agenda. Auch wird behandelt, wie Arbeit wohnortnah organisiert werden kann, um Berufstätigen mehr Zeit für mitmenschliche Beziehungen zu ermöglichen und Stress zu reduzieren, statt in überfüllten Zügen oder auf verstopften Autobahnen zu entfernten Arbeitsstätten zu pendeln oder für Arbeitsbelange stetig verfügbar zu sein [40]. Ebenso sind Konsequenzen der Digitalisierung von Arbeitsvorgängen und der automatisierten Entscheidungen für die mentale Gesundheit in der KoGeFö *mit* der Kommune relevanter, als die Bearbeitung typischer Handlungsfelder der individuellen Prävention und Gesundheitsförderung (z. B. Bewegung, Ernährung, Entspannung).

Damit das gelingt, müssen politische Gremien und kommunale Verwaltungseinheiten „Gesundheit“ als Politikfeld erkennen, es akzeptieren und zur Querschnittsaufgabe machen. Sie müssen Bedarfe und Assets analysieren, ein Leitbild entwickeln, mit zivilgesellschaftlichen Akteuren*innen koalieren und die Bewohner*innen über das Leitbild, die Programme und Maßnahmen mitentscheiden lassen (Partizipation), um die in der Kommune anstehenden Themen zu bearbeiten. „Health in all policies“ und „intersektorale Gesundheitsförderung“ sind einschlägige Stichworte für den integrierten, partizipativen Ansatz der KoGeFö *mit* der Kommune [24]. Gegenseitige Reserviertheiten von Verwaltungseinheiten und „versäulte“ kommunale Strukturen, die zu Machtkonflikten führen können, sowie bürokratisch-paternalistisches Agieren der Akteur*innen, die Partizipation verhindern, als auch Ziel- und Wertekonflikte zwischen kommunalen Interessensgruppen hemmen die Umsetzung des partizipativen, intersektoralen Zusammenwirkens [3, 16].

## „Gesundheit“ als Ziel des kommunalen Handelns

Das Akzeptieren des Politikfelds „Gesundheit“ legt mitnichten bereits das Ziel der KoGeFö *mit* der Kommune fest. Auch die eingangs qualifizierte „gemeindliche Resilienz“ lässt noch Konkretisierungen offen.

Ein Verständnis von „Gesundheit“, das in allen Lebenssituationen und für alle Lebenslagen gilt, existiert nicht. „Gesundheit“ ist ein relationales und mehrdimensionales Konzept [12], sozial konstruiert, abhängig von kulturellen Praktiken, Zielsetzungen und professionellem Handeln. Was unter Gesundheit verstanden wird, wie sie sich erhalten, wiederherstellen oder fördern lässt, unterliegt einem historischen und gesellschaftlichen Wandel. In vorsäkularen Kulturen wurde Gesundheit anders verstanden als in den heutigen säkularisierten Gesellschaften. Im Laienverständnis wird sie anders gefasst, als aus wissenschaftlicher Sicht.

Um im partizipativen Ansatz der KoGeFö *mit* der Kommune handlungsfähig zu werden, müssen die Proponent*innen ein gemeinsames Verständnis finden. Im Lichte der „großen gesellschaftlichen Herausforderungen“ ist weder mit einem Begriffsverständnis, das Gesundheit als Abwesenheit von Krankheit definiert, noch mit einem, das sich einseitig auf die körperliche oder seelische Gesundheit konzentriert (z. B. „Gesundheit ist der normgerechte Zustand der Organfunktionen“) oder mit einem, das Gesundheit umfassend und positiv als „vollkommenes Wohlbefinden“ definiert, ein operationalisierbares Ziel für die Aufgabe definiert, „gemeindliche Resilienz“ zu schaffen.

Für die Absicht, das Ziel der KoGeFö *mit* der Kommune über seinen Gebrauch, statt real zu definieren, leistet das sozial-ökologische Paradigma mehr, weil in ihm das komplexe Geschehen des kommunalen Handelns wirklichkeitsnäher abgebildet ist, als in biomedizinischen Ansätzen, die lineare Verursachungsketten für individuelle Gesundheitsstörungen postulieren. Das sozialökologische Fundament des im Folgenden unterbreiteten Vorschlags bezieht sich auf politisch-ethische Ansätze [26] und übernimmt gesundheitswissenschaftliche Perspektiven [43].

In der Praxis der Gesundheitsförderung wird häufig das „Regenbogenmodell“ von Dahlgren und Whitehead [9] bemüht. Das Modell ist deskriptiv, verweist auf die soziale Determination von Gesundheit, indem es illustriert, dass der gesundheitliche Status eines Individuums von Person- und Umweltfaktoren gleichermaßen beeinflusst wird. Die Nennung von Umwelt- und Personendeterminanten der Gesundheit ist aber noch keine sozialökologische Ausarbeitung. Für die KoGeFö *mit* der Kommune heute noch wichtige sozialökologische Modelle, die beschreiben und erklären, stammen u. a. von Glass und McAtee [13] oder Stokols, Allen und Bellingham [32]. Wegen der Konzentration auf das „embodiment“ und die „soziale Diskriminierung“, den beiden verwobenen Sachverhalten, dass sich soziale Phänomene in körperlichen und mentalen Zuständen manifestieren und umgekehrt, körperliche und mentale Zustände soziale Phänomene beeinflussen, und wegen der Suche nach Antworten auf die zentrale Frage, wer und was soziale Ungleichheiten in der Bevölkerungsgesundheit bedingt, ist auch die „ecosocial theory“ von Krieger [22] ein relevantes sozialökologisches Fundament für die GeFöKo *mit* der Kommune.

Im Kern postulieren sozialökologische Ansätze ein dynamisches Zusammenwirken von individuellen Fähigkeiten und Fertigkeiten, Selbstwirksamkeitserleben und Regulatoren in der sozialen und dinglichen Umwelt, das in einen Status verschiedener Gesundheitsdimensionen mündet: körperlich (z. B. Zustand der Organsysteme), psychisch (z. B. Zustand der kognitiven und emotionalen Prozesse), subjektiv (Wohlbefinden und Lebenszufriedenheit), funktional (z. B. sich selbst versorgen) und sozial (z. B. am sozialen Leben teilhaben). Der aktuelle Status wiederum beeinflusst die „Person x Umweltinteraktion“ [30]. Für Nussbaum [26] stehen Fähigkeiten – die sie „kombinierte Fähigkeiten“ nennt – für Handlungsmöglichkeiten einer Person („Tätigsein“), die sich aus dem Zusammenwirken von „personalen“ oder „internen Fähigkeiten“ und der Umweltmöglichkeiten ergeben. Aus Möglichkeiten wählen zu können, ist den „kombinierten Fähigkeiten“ inhärent.

Das regulative Potenzial der Umwelt zeigt sich – neben anderem – als „Umweltzugänglichkeit“, etwa in den Gelegenheiten, die sich bieten, den Alltag körperlich aktiv zu gestalten, sich gesund zu ernähren, am gesellschaftlichen Leben teilzuhaben oder andere Verhaltensweisen zu praktizieren, die das Wohlbefinden, die Lebenszufriedenheit und das persönliche Wachstum fördern. Umweltangebote oder -aufforderungen können ein Verhalten anregen, das vor Risiken schützt, individuelles Wachstum, Wohlbefinden und Lebenszufriedenheit fördert. Umwelt kann aber auch belasten, subjektiv beanspruchen und als stressend erlebt werden (z. B. Feinstaub, Lärm, Hitze, anhaltende Erreichbarkeit für berufliche Sachverhalte, gruppentypische Einstellungen). Sie kann gesundes Verhalten behindern respektive riskantes Verhalten fördern (rauchen, Alkoholabusus, wenig Schlaf etc.). Die Interaktion von Person- und Umweltfaktoren ist im „Sozialgradienten der Gesundheit“ typisch zu beobachten [19, 22]. Sozial marginalisierten Bevölkerungsgruppen fehlt es an gesundheitsförderlicher Umweltzugänglichkeit. Dort belastet Umwelt und verstärkt riskantes Verhalten. Dort wird Umwelt aber auch geschaffen, die ein ungesundes Verhalten nahelegt. Das zeigt sich beispielsweise in Studien zur „adipogenen Umwelt“ [23] oder zur „deprivation amplification“ [10]. Der Zusammenhang von Umwelt und individuellem Verhalten war bereits in den 1920er-Jahren Gegenstand der „Chicagoer Schule der Soziologie“.

## Die „gesunde Kommune“ als Ziel

Die KoGeFö *mit* der Kommune will die „gesunde Kommune“. Sie verfolgt das Ziel, die Kommune als Lebenswelt (ökologisch) resilient zu entwickeln und zu befähigen [4, 6]. Das Ziel kann sich am „Fähigkeitenansatz“ [26] orientieren. Kommunales Handeln soll ermöglichen, individuelle Lebenschancen wahrzunehmen. „Verwirklichungschancen“ ergeben sich bei Nussbaum [26] aus „internen“ und „kombinierten Fähigkeiten“ (Wahlfreiheiten: z. B. Gelegenheiten, Angebote zu nutzen oder nicht zu nutzen, Aufforderungen nachzukommen oder sie zu ignorieren), die es ermöglichen, ein Leben nach eigenen Bedürfnissen, Plänen und Absichten führen zu können. Dazu gehört neben anderen, sich vor Krankheiten zu schützen, soziale Kontakte zu pflegen, am gesellschaftlichen Leben teilzunehmen und persönlich wichtige Ziele verfolgen zu können. „Verwirklichungschancen“ ergeben sich aus der nicht auflösbaren Interaktion „interner Fähigkeiten“, der psychophysischen Konstitution, der materiellen Ausstattung einerseits und Möglichkeiten in der physischen, natürlichen und sozialen Umwelt (z. B. Einrichtungen, die Gesundheitsbildung ermöglichen; Mentalitäten, die das Verhalten nahelegen; Werte, die das Verhalten „gutheißen“) andererseits. Wenn beispielsweise sommerliche Hitzeperioden ältere Menschen hindern, ihre Wohnung zu verlassen, um sich mit anderen Personen zu treffen, Beziehungen zu knüpfen, Freundschaften zu pflegen und soziale Identität zu bewahren, dann sind ihre Verwirklichungschancen durch Umweltgegebenheiten eingeschränkt.

Die WHO etikettiert eine Kommune als „gesund“, wenn bei politischen und administrativen Entscheidungen abgewogen wird, welche Folgen die Entscheidungen für die Gesundheit und das Wohlergehen der Bürger*innen haben [4, 39]. Dieses Verständnis hebt auf den Prozess kommunaler Entscheidungsprozesse ab, die dem Gegenstand (Gesundheit und Wohlergehen) dienen. Damit ist aber weder der Endpunkt noch ist der „impact“ benannt, der über Programme und Maßnahmen der kommunalen Gesundheitsförderung angestrebt werden soll.

Angelehnt an den „Fähigkeitenansatz“ eröffnet eine „gesunde Kommune“ ihrer Bevölkerung Verwirklichungschancen, um gesund aufzuwachsen, älter zu werden, gesundheitliche Kompetenzen zu stärken und weitere Gesundheitsziele anzustreben. „Verwirklichungschancen“ ergeben sich, wenn Kommunen „Möglichkeitsräume“ für ein Verhalten schaffen, das den Gesundheitszielen dient. „Möglichkeitsräume“ sind in der Gerontologie, den Raumwissenschaften, der Stadtplanung, in den Bildungswissenschaften und in „Urban-Health-Ansätzen“ ein geläufiger Begriff für physische, soziale und mentale „Räume“, die ein (gesundheitlich) relevantes Verhalten ermöglichen und außerdem erleichtern und gutheißen.

Im Gutachten des „Wissenschaftlichen Beirats für Agrarpolitik, Ernährung und gesundheitlichen Verbraucherschutz beim Bundesministerium für Ernährung und Landwirtschaft“ [41] wird empfohlen‚ faire Umgebungen‘ zu schaffen. (Ernährungs)umgebungen gelten als ‚fair‘, wenn sie auf die Wahrnehmungs- und Entscheidungsmöglichkeiten sowie die Verhaltensweisen von Menschen abgestimmt sind, mehr Wahlmöglichkeiten bieten und es erleichtern, individuelle und gesellschaftliche Ziele zu verwirklichen. In Ansätzen der Gesundheitsförderung wie ‚make the healthy choice the easy choice‘ scheint diese Absicht auf [34].

**Abb. 1:**
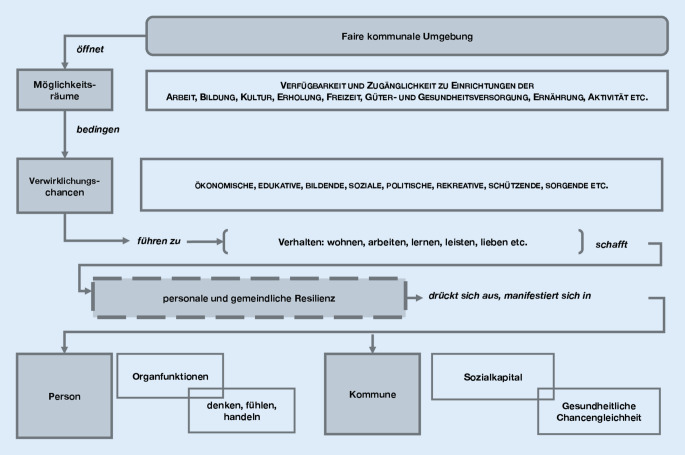
Illustration des Zusammenwirkens der Kommune als faire Umgebung mit Möglichkeitsräumen und Verwirklichungschancen

In Abb. 1 ist der Zusammenhang von „fairen Umgebungen“, „Möglichkeitsräumen“ und „Verwirklichungschancen“ illustriert.

Ziel der KoGeFö *mit* der Kommune ist, die Kommune „fair“ zu gestalten, um den Bewohner*innen „Möglichkeitsräume“ zu öffnen, die sie zu „Verwirklichungschancen“ nutzen können, um sich gesund zu verhalten und so nach Gesundheitszielen zu streben und sie mit höherer Wahrscheinlichkeit zu realisieren.

Konkrete Operationalisierungen (gegebenenfalls auch als normierte Messgrößen), die indizieren, wann „Fairness“ ausreichend gegeben ist oder Qualitäten, die das nachvollziehbar beschreiben, sind offen. Nussbaum [26] hat 10 zentrale Fähigkeiten benannt, die kommunale und staatliche Ordnung zu sichern hat. Normierte Größenvorgaben hat sie abgelehnt. In der KoGeFö *mit* der Kommune sind Endpunkte wie die Inzidenz oder die Prävalenz nicht-ansteckender Erkrankungen mögliche Indikatoren, um die Wirksamkeit von Interventionen zu beurteilen. Auch sind Messoperationen, die das „gute Leben“ abbilden und an die kommunale Lebenswelt angepasst wurden, geeignet. Dazu gehören z. B. „Glücksindizes“ wie sie im „World Happiness Report“ oder im „Better Life Index“ der OECD verwendet werden, sowie Messungen der Lebenszufriedenheit und des subjektiven Wohlbefindens. Ebenso wären Veränderungen in den Voraussetzungen der Verwirklichungschancen, der Fairness und der Zugänglichkeit zu, der Erleichterung von Wahlfreiheiten und das Gefühl der sozialen Zugehörigkeit als Indikatoren brauchbar. Statt die Wirksamkeit anhand nur eines Index zu beurteilen, sind in komplexen populationsbasierten Interventionen multiple Indikatorenbündel geeigneter. Schließlich sind methodische Konsequenzen zu diskutieren, da der Königsweg des Evidenznachweises, das RCT, die Komplexität der KoGeFö nicht angemessen abbilden kann.

Damit sich „Möglichkeitsräume“ öffnen, die sich für „Verwirklichungschancen“ nutzen lassen, müssen Bewohner*innen befähigt sein oder werden, ihre Chancen zu nutzen. Das verlangt nach Gesundheitsbildung, die Selbstwirksamkeit stärkt, Kompetenzen (Wissen und Können) entwickelt, um gesundheitliche Risiken realistisch einzuschätzen, Informationen zu gesundem respektive riskantem Verhalten zu verstehen und sie in Handlungen umzusetzen [15].

Gesundheitsförderung *mit* der Kommune verlangt zudem nach Ressourcenausstattung und Befähigung zur Implementierung von zielführenden Programmen und Maßnahmen. Für die finanzielle Ausstattung der Kommunen hat Burgi [5] „regionale Präventionsbudgets“ vorgeschlagen und deren Allokation an die Verpflichtung der Verwaltung zur integrierten Koordination und Kooperation mit zivilgesellschaftlichen Akteuren*innen geknüpft.

Zur Befähigung der kommunalen Akteur*innen könnten Studiengänge, die auf die transformativen Prozesse fokussieren, Personal qualifizieren, das mit dem vorhandenen Budget vor Ort „Wenden“ intersektoral initiiert, plant, überwacht und lenkt, die zur „gemeindlichen Resilienz“ führen. Auch der diskursive Austausch mit internationalen und nationalen Initiativen wie dem „Gesunde Städte Netzwerk“ bildete neue, stärkte vorhandene Kompetenzen und lieferte Anregungen für strukturelle und funktionale Veränderungen in Politik und Verwaltung.

## Fazit für die Praxis

- Die „gesunde Kommune“ zu entwickeln, ist ein komplexes Unterfangen, das in einem intersektoralen und partizipativen Prozess schrittweise vorgeht.
- Am Anfang gilt es, die kommunale Umgebung hinsichtlich ihrer „Fairness“ zu analysieren, also alle politischen und administrativen Entscheidungen (Siedlungsbau, Verkehr, Bildung, Betreuung, Sicherheit etc.) – im Sinne des Health-in-all-policies-/ all-government-Ansatzes – in ihrer gesundheitlichen Konsequenz und in der Passung zur gesellschaftlichen Transformation (z. B. das UN-Nachhaltigkeitsziel Nr. 3) zu hinterfragen.
- Die Umgebung der Kommune dann – im Lichte der Befunde – in einem partizipativen Prozess zwischen Politik, Verwaltung und Zivilgesellschaft fair(er) zu gestalten.
- Den Bewohner*innen sodann „Möglichkeitsräume“ (Angebote schaffen, Zugänglichkeit erleichtern etc.) zu öffnen und sie so zu ertüchtigen und zu motivieren, ihre Chancen zu verwirklichen, um ihre persönlichen Ziele zu verfolgen.
